# Strategies of the honeybee *Apis mellifera* during visual search for vertical targets presented at various heights: a role for spatial attention?

**DOI:** 10.12688/f1000research.4799.1

**Published:** 2014-07-28

**Authors:** Linde Morawetz, Lars Chittka, Johannes Spaethe

**Affiliations:** 1 Department of Integrative Zoology, University of Vienna, Vienna, 1090, Austria; 2 Behavioral Physiology and Sociobiology, Biozentrum, University of Würzburg, Würzburg, 97074, Germany; 3 Biological and Experimental Psychology, School of Biological and Chemical Sciences, Queen Mary University of London, London, E1 4NS, UK

## Abstract

When honeybees are presented with a colour discrimination task, they tend to choose swiftly and accurately when objects are presented in the ventral part of their frontal visual field. In contrast, poor performance is observed when objects appear in the dorsal part. Here we investigate if this asymmetry is caused by fixed search patterns or if bees can use alternative search mechanisms such as spatial attention, which allows flexible focusing on different areas of the visual field.

We asked individual honeybees to choose an orange rewarded target among blue distractors. Target and distractors were presented in the ventral visual field, the dorsal field or both. Bees presented with targets in the ventral visual field consistently had the highest search efficiency, with rapid decisions, high accuracy and direct flight paths. In contrast, search performance for dorsally located targets was inaccurate and slow at the beginning of the test phase, but bees increased their search performance significantly after a few learning trials: they found the target faster, made fewer errors and flew in a straight line towards the target. However, bees needed thrice as long to improve the search for a dorsally located target when the target’s position changed randomly between the ventral and the dorsal visual field. We propose that honeybees form expectations of the location of the target’s appearance and adapt their search strategy accordingly. Different possible mechanisms of this behavioural adaptation are discussed.

## Introduction

When honeybees search for targets presented on a vertical plane, they show a distinct spatial asymmetry in colour and pattern learning between the ventral and the dorsal half of their frontal visual field. They easily learn a target when its defining features are perceived by the ventro-frontal area of the eye, but are less accurate when the crucial features of the target appear in the dorso-frontal area ( Baumgärtner, 1928; Chittka *et al.*, 1988; Giurfa *et al.*, 1999; Lehrer, 1999; Morawetz & Spaethe, 2012; Wehner, 1972). This behavioural asymmetry might in theory be explained by specialization in eye morphology. This is found in the eye of the honeybee drone, where the dorsal area is adapted to queen detection by increasing visual acuity and sensitivity, which is partly achieved by enlarged facet diameters and a reduction of the interommatidial angles ( Menzel *et al.*, 1991; Seidl, 1982; Streinzer *et al.*, 2013; van Praagh *et al.*, 1980). However, such regional specialisation in eye optics is not found in the worker honeybee, where the interommatidial angles and facet diameters are similar in the frontal visual field 30° below and above the horizontal plane ( Seidl, 1982).

It is also possible that regional specialisation of the visual system occurs at the neuronal level. Indeed, ascending neurons of the medulla show differences in arborisation patterns between the ventral and dorsal area ( Ehmer & Gronenberg, 2002), indicating that both areas of the visual field are to some extent processed separately. Furthermore, the output of these two areas becomes segregated in the anterior optic tubercle and in the collar of the calyxes of the mushroom bodies ( Ehmer & Gronenberg, 2002; Mota *et al.*, 2011). The data hint at differences in neuronal processing between these two eye regions and correspond with the behavioural evidence of a dorso-ventral differentiation ( Baumgärtner, 1928; Chittka *et al.*, 1988; Giurfa *et al.*, 1999; Lehrer, 1999; Morawetz & Spaethe, 2012; Wehner, 1972).

An alternative explanation for the dorso-ventral asymmetry is the usage of attentional mechanisms, which focuses the visual processing capacity of the brain flexibly to the currently most important area in space (spatial attention; Carrasco & McElree, 2001; Druker & Anderson, 2010; Geng & Behrmann, 2002; Posner, 1980; Yantis & Jonides, 1984). Attention here can be thought of as a kind of ‘inner’ eye, focusing on a spatial subset of the information that is available from the visual sensory periphery. The existence of spatial attention is well known in vertebrates, but has only recently been described for the first time in insects, the fruit fly *Drosophila melanogaster* ( Sareen *et al.*, 2011) and the honeybee *Apis mellifera* ( Paulk *et al.*, 2014).

Spatial attention optimizes search processes in detection tasks, where the subject has an expectation of the appearance of the object, using external cueing or own experience ( Posner, 1980). Search efforts can then be directed to this region which leads to faster and more accurate decisions ( Carrasco & McElree, 2001). Hence, spatial attention would be a useful tool for foraging bees helping to adapt to various spatial settings of flower distribution. The dorso-ventral asymmetry observed in visual discrimination tasks could be explained by attention being focused on the ventral part of the visual field by default, but the attentional focus could be moved to other parts of the visual field, if necessary. To test if bees can employ spatial attention during foraging, we confronted honeybees with three search scenarios differing in the positioning of the target in the visual field. This approach allows to analyse the changes in search performance and flight behaviour over time and therefore to identify possible adaptations of the search behaviour to the particular target presentation.

## Material and methods

The experiments were conducted between July and September in 2011 on the terraces of the Biozentrum, University of Vienna, where several hives of *Apis mellifera* were located. Bees were trained to an experimental box and marked individually.

### Experimental setup

**Figure 1.**
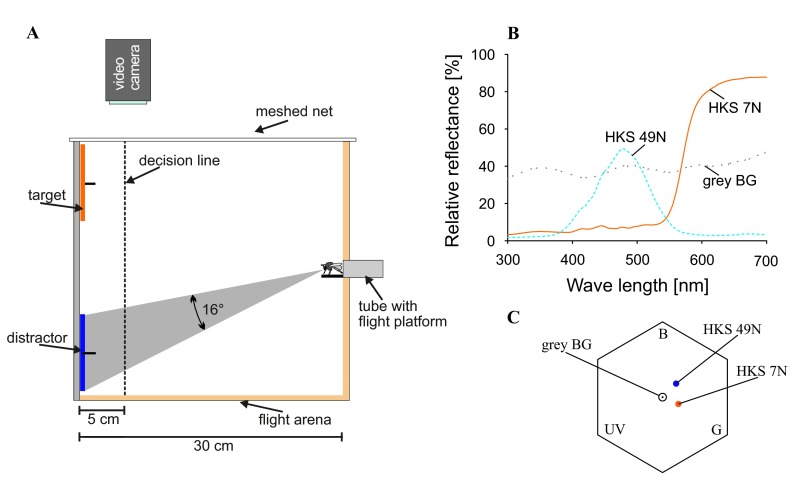
Experimental setup and colorimetry. **A**. Side view of the experimental arena. Bees enter the arena through a Plexiglas tube, view the objects on the back wall and fly towards one of them. The bee’s decision is counted as correct when the bee crosses the decision line (5 cm in front of the back wall) at the position of the target. The bee’s flight can be observed and filmed through a small-meshed net from above and through a Plexiglas cover from the side. The number gives the angle of the target subtended on the bee’s eye when viewed from the entrance of the box. **B**. Reflectance curves of the back wall cover (grey BG - background), the orange target (HKS 7N) and the blue distractor (HKS 49N). **C**. Colour hexagon showing the colour loci of the target and the distractor (colour distance of HKS 7N to background: 0.23 hexagon units; HKS 49N to background: 0.25 hexagon units; distance between the two colours: 0.27 hexagon units); calculations after Chittka (1992).

A wooden box (30 × 54 × 40 cm) served as experimental arena ( Figure 1A; see also Morawetz & Spaethe, 2012). The bees could enter the box through a Plexiglas tube on the front and shutters in the tube allowed to control access to the box. Two video cameras (DCR-SR55, Sony, Minato, Tokyo, Japan) were placed above and at one side of the box to record the flights through a small-meshed net and a Plexiglas wall, respectively.

The back wall was divided into nine fields (3 rows by 3 columns) of which only the top and the bottom row were used for training and testing. In the centre of each field a platform provided reward for a correct choice (1 M sucrose solution ad libitum) and punishment for an incorrect choice (0.1% quinine solution w/w; Chittka *et al.*, 2003), respectively. Stimulus discs (K + E, Stuttgart-Feuerbach, Germany) of 9 cm diameter were cut from coloured paper; the target discs were orange (HKS 7N) and distractor discs were blue (HKS 49N, for colour details see Figure 1B,C). The stimuli subtended a visual angle of >15° on the eye of the bee when sitting at the box entrance, which enabled the bee to perceive chromatic information from the beginning of the search ( Dyer *et al.*, 2008; Giurfa *et al.*, 1996). The stimuli were presented on the back wall of the arena subtending a visual field of 82° in the horizontal and 60° in the vertical on the eye of a bee ( Figure 1A). Stimuli located in the dorsal row were thus perceived by the dorsal part of the eye, while objects in the ventral row were perceived by the ventral part of the eye, when the bee entered the arena ( Figure 1A).

### Training and testing

Bees were trained to enter the experimental arena and to search for the orange target on the back wall. During this pre-training, special care was taken in presenting the target at different heights and positions in the experimental arena to avoid any position learning. After the first successful visit of the target, a training phase of 20 visits followed. The position of the target was changed in a pseudo-random pattern: on average, in 50% of the visits the target was presented in the top row, and in the other 50% in the bottom row. The target discs and feeders were exchanged with clean discs/feeders after every third visit to avoid odour contamination.

**Figure 2.**
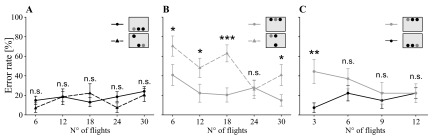
Search accuracy of honeybees depending on treatments. Treatments differed in the positioning of the rewarding target and distractors which were associated with a punishment (insets: grey circle = target, black circle = distractor). In the ‘dorsal group’ (straight grey line, grey circle) the target was always presented in the top row, in the ‘ventral group’ (straight black line, black circle) it was always located in the bottom row, and in the ‘mixed group’ the position of the target alternated randomly between both rows (top row: dashed grey line, grey triangle; bottom row: dashed black line, black triangle). **A**, **B**. Comparison between the one row condition and the two row condition separated in trials where the target appears in the bottom row ( **A**) and trials with the target in the top row ( **B**). **C**. Comparison of the first 12 decisions of the dorsal group and the ‘ventral group’. Statistics: general linear model with binomial distribution: n.s. P>0.10, * P<0.05, ** P<0.01, ***P<0.001; data points: mean ± s.e.m., N=9 individuals per group.

The training phase was followed by an experimental phase, which consisted of 30 flights (five blocks, six flights per block). Bees were divided in three experimental groups. Bees from all groups had to search for one orange target among two blue distractors, but the groups differed in the placement of the objects (see insets of Figure 2). In the ‘dorsal group’, all objects were always placed in the top row; in the ‘ventral group’, they were only presented in the bottom row. In the ‘mixed group’, target and distractors could appear in the top row as well as in the bottom row. The target position was changed in a pseudo-random order assuring that the target was presented 50% of the trials in the top row and 50% of the trials in the bottom row. The distractor positions were changed randomly between the remaining five positions of the two rows. Therefore, bees of the ‘dorsal’ and ‘ventral group’ needed to search only in a subarea of the search field (three possible positions), while bees of the ‘mixed group’ had to search the target within the entire search area (six possible positions).

### Data analysis and statistics

Search efficiency was measured using two parameters – error rate and decision time. This allowed to check for possible speed-accuracy trade-offs ( Burns & Dyer, 2008; Chittka *et al.*, 2003). We counted a bee’s choice when it crossed a decision line, which was indicated by a grid of white twine 5 cm in front of the back wall of the experimental box ( Figure 1; Morawetz & Spaethe, 2012). We counted an error when bees crossed this decision line at a location without a target. The decision time was defined as the time the bees needed from entering the box until they crossed the decision line.

We analysed the video recording using The Observer XT Version 7 (Noldus, Wageningen, The Netherlands) and reconstructed the flight paths of the bees using SkillSpector 1.3.0. (Video4coach, Svendborg, Denmark). Statistics was calculated using the statistical package R version 2.14.1 ( R Development Core Team, 2011). We used mixed linear models to test the effect of treatment group, target location and learning on the error rate and on the decision time, respectively. Furthermore, the identity of the individual bees was implemented into the model as a random factor. For analysing the error data, a binomial distribution was applied using the function *lmer* of the package ‘lme4’ ( Bates *et al.*, 2011), while for the decision time, a normal distribution was applied using the same function. Significance levels were determined by calculating a type-III ANOVA using the function *Anova* (package ‘car’, Fox & Weisberg, 2011). To check if the bees showed a general tendency to fly upwards or downwards during the different flight stages, the flight angles were tested against 0° (horizontal flight) with a Wilcoxon test. Figures were created using Sigma Plot 11.0 (Systat Software Inc., San Jose, USA) and Corel Draw X3 (Corel Corporation, Ottawa, Canada).

## Results

### Error rate

During the training phase, when only the target was present, bees of all treatment groups showed similar accuracy (χ ^2^_(2)_=1.37, P=0.502). At the end of the training, the bees made four times fewer mistakes when searching for ventrally located targets compared to targets located in the dorsal row (bottom row: 15% ± 25% s.d.; top row: 65% ± 33%; χ ^2^_(1)_=34.32, P<0.001; see also dataset 1). During the test phase, the overall error rate was similar in all three treatment groups (χ ^2^_(2)_=2.79, P=0.248; see also dataset 2), but the speed of improvement differed between the three groups (interaction treatment group x learning block: χ ^2^_(8)_=19.58, P=0.012).

Bees of the group presented with targets in the ventral visual field showed a constant performance during the test with a mean error rate of 17% ± 14 s.d. (χ ^2^_(4)_=2.72, P=0.606; Figure 2A, straight line). Conversely, when bees were presented with targets in the dorsal visual field, they made about twice as much mistakes at the beginning of the test than at the end (χ ^2^_(4)_=12.84, P=0.012; Figure 2B, straight line). However, the overall error rate did not differ from the ‘ventral group’ (χ ^2^_(1)_=0.78, P=0.377; Figure 2A). The main change in learning performance in the ‘dorsal group’ took place between the first and second learning block; thus a possible difference between both treatments at the beginning of the test phase may have been masked. We then analysed the first 12 decisions in more detail, subdividing the 12 visits into blocks of three ( Figure 2C). During the first three flights, bees of the ‘dorsal group’ made four times more mistakes than bees of the ‘ventral group’ (z = 2.77, P=0.006; Figure 2C). After this initial phase, both groups achieved similar levels of accuracy (all further learning blocks: P>0.05; Figure 2C).

The ‘mixed group’ was the only group in which the target and distractors were presented in both the ventral and the dorsal part of the visual field. Similar to the ‘dorsal group’, bees improved their accuracy during the test (χ ^2^_(4)_=9.54, P=0.049). Bees of the ‘mixed group’ made fewer mistakes when the target was presented in the ventral row compared to the situation when it was presented in the dorsal row (χ ^2^_(1)_=5.27, P=0.022; Figure 2A,B dashed lines). Next, we analysed both data sets separately and compared them to the correspondent single row treatment group ( Figure 2A: flights of the ‘mixed group’ when the target was ventrally presented compared to the ‘ventral group’; Figure 2B: flights of the ‘mixed group’ when target was dorsally presented compared to the ‘dorsal group’). The performance of bees from the ‘mixed group’, when searching for a ventral target, was similar to the performance of the ‘ventral group’ where the target was always presented ventrally (χ ^2^_(1)_=0.35, P=0.556; Figure 2A). Furthermore, bees of the ‘mixed group’, when searching for ventral targets, showed a constant performance during the experiment (χ ^2^_(4)_=3.90, P=0.420). However, they made significantly more mistakes than bees of the ‘dorsal group’ in finding a dorsally positioned target (χ ^2^_(1)_=7.69, P=0.006; Figure 2B). Nonetheless, the ‘mixed group’ improved their performance for dorsal targets (χ ^2^_(4)_=14.05, P=0.007), and in the fourth learning block, bees managed to achieve a similarly low error rate as the bees of the ‘dorsal group’ ( Figure 2B).

### Decision time

**Figure 3.**
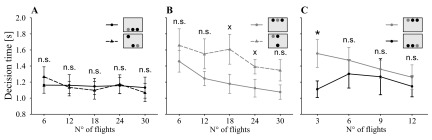
Decision time of honeybees depending on treatments. Decision time of the three experimental groups: ‘ventral group’ (black straight line, black circle), ‘dorsal group’ (grey straight line, grey circle) and ‘mixed group’ (ventral half: black dashed line, black triangle; dorsal half: grey dashed line, grey triangle). **A**. When searching for the target in the ventral half of the visual field, the bees of the ‘mixed group’ made their decision at a similar speed as the ‘ventral group’ (χ ^2^_(1)_=0.00, P=0.979). They did not improve their performance (χ ^2^_(4)_=2.25, P=0.689). **B**. Comparing the ‘dorsal group’ with the dorsal flights of the ‘mixed group’, the bees showed no significant difference in their decision time (χ ^2^_(1)_=3.02, P=0.082). All bees became faster in the course of the experiment (χ ^2^_(4)_=22.84, P=0.001). **C**. Comparison of the ‘dorsal’ and the ‘ventral group’. Only during the first three decisions the ‘dorsal group’ was significantly slower than the ‘ventral group’ (t _(13.10)_=2.211, P=0.045), afterwards bees of both groups had similar decision times (all other data points P>0.05). Statistics: t test: n.s. P>0.10, x P<0.10, * P<0.05; data points: mean ± s.e.m., N=9 per group.

All three experimental groups became faster during the experiment (χ ^2^_(4)_=16.83, P=0.002; Figure 3), but the decision time did not differ among treatment groups (χ ^2^_(2)_=1.04, P=0.595) or in the interaction of both factors (χ ^2^_(8)_=10.00, P=0.265). However, bees of the ‘mixed group’ were slower when the target was presented in the top row than when it was presented in the bottom row (χ ^2^_(1)_=28.6, P<0.001). Bees of the ‘dorsal’ and ‘ventral group’, on the other hand, showed similar decision times during most of the time (χ ^2^_(1)_=0.24, P=0.624). However, during the first three flights, bees of the ‘dorsal group’ were significantly slower than bees of the ‘ventral group’ (t _(13.1)_=2.21, P=0.045; Figure 3C).

### Flight path

**Figure 4.**
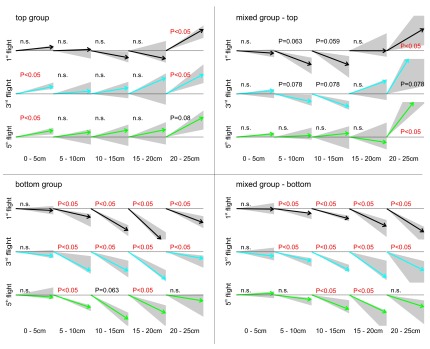
Flight structure of the three treatment groups. Median flight angles of different phases in the bee’s flights crossing the experimental arena from the entrance (0 cm) to the decision line (25 cm). For all four search situations the first (black arrows), the third (blue arrows) and the fifth flight (green arrows) are shown (N=7). Please note that in the mixed group the flight number refers to number of trials absolved with the particular target situation and not to the total number of trials the bee has absolved. Arrows: median flight angles; grey area: first to third quartile; statistics: one-sample Wilcoxon test against 0° (flight without significant trend of changing flight height).

When analysing the flight structure of the first flights in detail, it became evident that bees of the ‘dorsal group’ increased the steepness of their departure angle (flight angle between 0 and 5 cm distance from the entrance) significantly from the first to the third flight ( Figure 4; Wilcoxon test between first and third flight: P<0.05). During their first flight, they started horizontally into the box (median flight angle 5.9°, Figure 4), but from the third flight on they flew upwards when taking off (median flight angle third flight: 12.1° upwards; fifth flight: 10.0° upwards). This change in flight structure indicates that the bees decided earlier to fly upwards in their third flight compared to their first flight. All other experimental groups showed median departure angles between 7° downwards and 5° upwards – no significant trend in flying up- or downwards during the departure was found.

**Figure 5.**
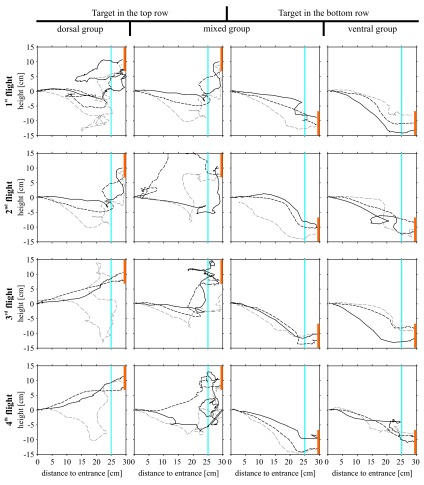
Flight paths of selected bees. The flight paths describe the differences in flight behaviour among the three experimental groups. The 16 squares represent cross sections of the flight box with the bee entering the box from the left side searching for the target (presented on the right side; orange rectangle). The blue line 25 cm away from the entrance marks the decision line. For each experimental group three individual bees are shown (straight black line, dashed black line, dashed grey line).

When the target was located in the bottom tier, the bees’ flight was composed of downward angles during most of their flight path, suggesting a continuous downward flight ( Figure 4, Figure 5). Bees searching for a dorsally located target, however, differed in their flight structure between treatment groups: bees of the ‘mixed group’ flew in a steep angle downwards during the first flight period (maximum flight angle 40° downwards) and showed a significant upwards direction between 20 and 25 cm distance from the entrance (median flight angle between 33° and 58° upwards, Figure 4). During the fifth flight of the experiment with the target in the top row, these bees lost their tendency to fly downwards at the beginning of their flight and flew straight forward for the first 20 cm and ascended steeply afterwards. Bees of the ‘dorsal group’ flew horizontally between the first 5 to 20 cm, with some animals already beginning to ascend. Between 20 and 25 cm they ascended in a steep angle of around 25° similar to the animals of the ‘mixed group’. Therefore, all bees searching for a top row target approached the target from below. Flight paths of individual bees demonstrate the described differences of the three different groups ( Figure 5).

In conclusion, the accuracy, decision time and flight path of bees searching for a vertically presented target was partly dependent on the target’s location and partly on the bees’ possibility to predict the target’s location. Searching for ventrally located targets was an easy task – all bees showed high accuracy, short decision times and a direct flight path. The predictability became important in situations in which bees had to detect the target with the dorsal part of their eyes. When the target was always in the top row (‘dorsal group’), bees adapted their search strategy rapidly to the situation –after three search flights they detected the target swiftly, decided accurately and approached the target directly. When the target was positioned randomly in the top or the bottom row (‘mixed group’), the bees showed a more downward directed flight pattern when being confronted with dorsally located targets. As a consequence, these bees took longer in making their decisions and made more mistakes.

Datasets for strategies of Apis mellifera during visual search for vertical targets Dataset 1: The dataset contains the decisions of each bee (correct: number of correct decisions; false: number of false decisions) during training phase. Data are divided in treatment groups (dorsal, ventral, mixed) and learning blocks of five trials (B1: trial number 1 to5; B2: trial number 6 to 10, etc.). The total number of decisions is given (_all) as well as the separation into trials with the target placed in the top row (_T_dorsal) and in the bottom row (_T_ventral).
Dataset 2: The dataset contains the decision time (seconds) and error rate (correct: number of correct decisions; false: number of false decisions) of each bee during the test phase. Data are divided in treatment groups (dorsal, ventral, mixed) and learning blocks of six trials (B1: trial number 1 to6; B2: trial number 7 to 12, etc.). For both error rate and decision time the total values are given (_all) as well as the separation into trials with the target placed in the top row (_T_dorsal) and in the bottom row (_T_dorsal). NA = missing value.
Dataset 3: The dataset contains the decision time (seconds) and error rate (correct: number of correct decisions; false: number of false decisions) of each bee during test the phase. Data are divided in treatment groups (dorsal, ventral, mixed) and learning blocks of three trials (B1: trial number 1 to 3; B2: trial number 4 to 6, etc.). For both error rate and decision time only the total values are given (_all).
Dataset 4: The dataset contains the flight angles of the first (F1), third (F3) and fifth (F5) trial of all filmed bees (N=7 per treatment group). For the mixed group these trials are presented for both possible target positions (dorsal, ventral) – therefore in this group 10 flights per bee are shown. The distance between entrance hole and decision line (25cm) was divided into 5 segments of 5 cm length (_5: 0 to 5 cm distance from the entrance, _10: from 5 to 10 cm distance, etc. ). NA = missing value).

## Discussion

We simulated three foraging situations that a bee might encounter in her natural environment: approaching flowers from above (flowers perceived by the ventral part of the eye), foraging within a 3D floral environment, either in a tree or a meadow with flowers at various heights (flowers may appear in the dorsal and ventral part of the visual field) and approaching a flower from below when foraging, for example, upwards on vertical inflorescences (flowers perceived by the dorsal area of the eye). We demonstrated that bees were able to adapt their detection strategy to all three foraging situations, although the course of performance improvement differs among the experimental conditions. In the following paragraphs, we discuss the possible proximate and ultimate mechanisms of this behavioural flexibility.

### ‘Ventral group’

Bees of this group were presented with one target and two distractors in the bottom row. They decided accurately and swiftly and had a downwards directed flight vector. It is therefore possible that bees, by default, focus on the ventral part of the frontal visual field when being confronted with vertically presented objects, a behaviour which might explain the dorso-ventral asymmetries in visual discrimination observed by several independent studies ( Baumgärtner, 1928; Chittka *et al.*, 1988; Giurfa *et al.*, 1999; Lehrer, 1999; Morawetz & Spaethe, 2012; Wehner, 1972).

### ‘Dorsal group’

In the ‘dorsal group’, both target and distractors were presented in the top row. During the initial flights, bees’ search was inaccurate and slow compared to the ‘ventral group’. The bees flew straight ahead for the first 20 cm in the flight arena and then turned upwards in a steep angle during the final approach. After the first three flights, bees showed two major changes in their behaviour: (1) the error rate dropped from 40 to 20% ( Figure 2C) and (2) they changed their departure angle from horizontal to, on average, 11° upwards ( Figure 4), a behaviour which was exclusively shown by the ‘dorsal group’.

The reason for the bees’ improvement might be explained by two independent but not exclusive mechanisms. Firstly, a top-down attentional mechanism could have accelerated information processing from the dorsal part of the frontal visual field by shifting the attentional focus to this area. In this case, bees might have detected the target stimulus just after entering the experimental arena and started an upward flight towards the top row. Alternatively, bees could have learned to adjust their flight path to the area where they expected the target: they learned to initiate an upward flight immediately after entering the arena before detecting the target. By flying upwards they were able to analyse the top row with the ventral part of their eye and therefore increase their accuracy. Evidence for both mechanisms comes from earlier studies: bees can learn to perform directional changes and changing flight routes to optimize foraging success ( Cheng & Wignall, 2006; Lihoreau *et al.*, 2010; Perry & Barron, 2013), as well as use attentional processes to selectively react to visual stimuli ( Giurfa *et al.*, 1999; Paulk *et al.*, 2014; Spaethe *et al.*, 2006). However, our results do not allow discriminating between both mechanisms.

### ‘Mixed group’

Bees of the mixed group were confronted with one target and two distractors, which were randomly distributed between the bottom and top row. During most trials, objects were located in both the bottom and the top row. Hence, the search strategy of the ‘mixed group’ was fundamentally different from the two other groups. Bees were not simply able to fly towards a group of objects, but had to decide which group of objects was relevant for them, i.e. contained the target. At the beginning of the experiment, bees flew downwards regardless of the target position – probably executing their ‘default’ behavioural pattern and flying towards the lowermost objects. This behaviour resulted in a low error rate when the target showed up in the bottom row, but in a high error rate and long decision times when the target appeared in the top row. Furthermore, our data indicate that bees detected ventrally located stimuli first, but analysed the chromatic features of the stimuli only later during flight.

Bees of the mixed group had to change their flight pattern to improve the search for targets located in the top row. A downwards orientated flight route can hamper the detection of a dorsally located target due to an unfavourable angle of view for objects in the top row. Additionally, it makes complex course corrections necessary (demonstrated by the flight curves in Figure 5). Indeed, bees of the ‘mixed group’ stopped flying downwards in the first part of their flight after their tenth trial ( Figure 4) and instead flew straight before deciding for an upwards or downwards movement.

Additionally, bees of the mixed group may have also applied mechanisms of visual attention to increase the detection performance of targets located in the top row. For example, bees could have increased the area of their attentional focus to a size large enough to cover both rows. This would have allowed the bees to process all presented objects simultaneously ( Eriksen & St. James, 1986; Pashler, 1987). In humans, an increase in the size of processing focus is normally accompanied by a decrease of processing quality ( Castiello & Umiltà, 1990; Eriksen & St. James, 1986). We did not find a decrease of performance level in the search for ventrally located target in the ‘mixed group’, which makes this explanation unlikely. An alternative search mechanism might be that bees learn to move their attentional focus from the ventral part of the visual field to the dorsal part after they had not detected a target in the ventral part. This explanation fits with the constant high search performance for targets in the bottom row and with longer decision times and late upward directed flight vectors, when searching for a target in the top row. The high error rate in the first part of the experiment probably owes to the learning process, in which the bees (1) learned to avoid flying downwards immediately after entering the arena and (2) learned to continue searching the top row when the target could not be detected in the bottom row.

### Ecological relevance

When searching for a flower patch, bees often approach the meadow or tree from above ( Kevan, 1990; Lehrer, 1999) – a situation represented by the conditions of the ‘ventral group’. Bees seem especially capable to deal with this type of search, as they detect ventrally positioned targets more easily ( Figure 2A; Lehrer, 1999; Morawetz & Spaethe, 2012; Skorupski *et al.*, 2006). However, when bees are foraging in a meadow with flowers at various heights, flowers can also appear in the upper part of the visual field and information received from the dorsal part of the eyes become important. For example, bees which visit raceme inflorescences tend to begin collecting nectar at the bottom of the inflorescence and ascend vertically step by step ( Fisogni *et al.*, 2011; Ishii *et al.*, 2008; Pyke, 1978; Valtueña *et al.*, 2013; Waddington & Heinrich, 1979). In this situation the flowers below the just probed ones, which are perceived by the ventral visual field, typically contain no nectar, because the bee had just visited them ( Heinrich, 1975; Ishii *et al.*, 2008). To optimize foraging efficiency, bees must focus visual processing on the dorsal part of the visual field or adopt an upward directed motor pattern. We demonstrated that bees can adapt to the described situation, improving their detection ability of dorsally located targets within three visits only ( Figure 2C).

When foraging in a blooming tree, a bee can expect a rewarding flower appearing in the ventral and the dorsal visual field with a similar probability. Flowers are densely distributed in a tree and bees normally see several flowers simultaneously. Interestingly, large bees as *Bombus* and *Xylocopa* reveal a complex pattern of upwards and downward movements inside a tree: when moving between neighbouring flowers, they move upwards, similar to the movement pattern on a vertical inflorescence ( Kevan, 1990). However, when moving to an inflorescence at distance larger than 20 cm they tended to fly downwards ( Kevan, 1990). This behaviour matches our observation that bees tend to fly to the lowermost object when approaching from a distance. However, we also demonstrated that bees can overcome this motor pattern within 10 learning trials, when it hinders the bees’ foraging success. Likewise, Kevan (1990) described that movement patterns of bees differed between tree species with different flower distributions and suggested that bees optimize their foraging strategy to the particular resource distribution.

In conclusion, we showed that bees flexibly adapt to a given foraging situation by focusing their detection and discrimination effort to the appearance of the object within their visual field. They probably use both attentional mechanisms and behavioural strategies to optimize their foraging success, although more data are needed to clearly separate between these mechanisms. This flexibility provides the ability to choose among different search strategies and to quickly adapt to various foraging environments.

## Data availability

***figshare***: Datasets for strategies of *Apis mellifera* during visual search for vertical targets, doi: http://dx.doi.org/10.6084/m9.figshare.1104387 ( Morawetz *et al.*, 2014).
