# Supplementary material for: Strategies of the honeybee Apis mellifera during visual search for vertical targets presented at various heights: a role for spatial attention?
Source: F1000Res. 2014 Jul 28;3:174. [Version 1] doi: 10.12688/f1000research.4799.1 (PMC4168805; doi:10.12688/f1000research.4799.1)
Supplement: Peer review history

# Peer review: Referee response for version 1

**DOI:** 10.5256/f1000research.5123.r5608

**Published:** 2014-09-09

**Authors:** Martin Giurfa

**Affiliations:** 1 Research Center on Animal Cognition, Centre National de la Recherche Scientifique (CNRS), Toulouse, France

This paper analyzes visual target recognition in bees and focuses on previously reported asymmetries between the dorsal and the ventral visual fields in terms of color/pattern recognition and discrimination. The authors studied whether a pattern detection/discrimination task varies when patterns are displayed either in the ventral or in the dorsal visual fields and discuss different possible mechanisms accounting for this variation. Specifically, bees were trained to choose a rewarded orange target which was presented among blue distracters either in the ventral visual field ('ventral' group' of bees), the dorsal field ('dorsal' group') or in both fields ('mixed group'), and choice errors, decision time, and flight pathways upon entering into the experimental arena were recorded.

The authors show in a **convincing way **that bees presented with targets in the ventral visual field were most efficient in detecting the rewarded target as they made rapid decisions with high accuracy and direct flight paths. In contrast, bees perceiving the patterns in the dorsal visual field made more errors and took more time to detect the pattern. Yet these 'dorsal bees' improved significantly their performance after a few learning trials as they decreased both the number of errors and decision time and flew in a straight line towards the target.  Bees of the 'mixed group' needed considerably more time to improve the search of the target whose position changed randomly between the ventral and the dorsal visual field.

The conclusions drawn by the authors focus on the notion of expectation as it is said that "bees form expectations of the location of the target’s appearance and adapt their search strategy accordingly". Furthermore, the authors insist on the notion of attention (more specifically, spatial attention), which is certainly attractive in the light of the results obtained, but as the authors themselves acknowledge, ***is probably not the unique explanation for their data***.

As a consequence, a more suitable title for the paper, given the results presented, would be

something like: " *Honey bees exhibit flexible (plastic) visual search strategies for targets presented vertically at various heights*". It seems to me that the main finding of the paper is precisely the flexibility and adaptability of the bees' visual search strategies to the various experimental situations rather than the proof of attentional processes guiding these strategies.

A **main concern** raised by this paper relates to the criteria (unclear to me) used to define the position of the so-called **"decision line"** in the setup. Clearly such a line was very close (5 cm) to the targets so that decision counting may have under/over estimated real decisions performed (and perhaps) modified at further distances from the targets. It would be therefore interesting to contrast the decisions counted and established following the authors' method with a different one, which, for instance, may be based on quantifying angular deviations of trajectories along the flight path; one can establish a criterion for a decision based on an angular deviation from the entrance vector and quantify decision which may occur before reaching the decision line defined by the authors.

A **minor concern** relates to incomplete citations in the paper. In particular, given that the focus is on ventral/dorsal asymmetry of visual performances on bees, it would be worth considering and mentioning the work on ventral vs. dorsal target detection in bees ( Giurfa *et al*., 1999) . Additionally, the statement on the relative recency of studies on attention in insects is questionable. Although it is not explicitly said - probably because of constraints imposed by reviewers - the work by van Swinderen and Greenspan (2003) constitutes a study on visual attention. Also, if one focuses on bees, the use of this concept of attention dates to 2004 ( Giurfa, 2004) when it was argued that discrimination between perceptually closer stimuli was possible after differential conditioning but not after absolute conditioning due to differences in attention inculcated by these two training procedures. The idea was further developed in later papers, so that referring the use of this concept in insects to 2011 seems a bit unfair to prior contributions which also focused on the same idea.

All in all, the manuscript is sound and well-written. The results are quite convincing (but see remark on the appropriateness of the decision line criterion) and constitute an important contribution to field of insect vision.

I have read this submission. I believe that I have an appropriate level of expertise to confirm that it is of an acceptable scientific standard.

# Peer review: Referee response for version 1

**DOI:** 10.5256/f1000research.5123.r5610

**Published:** 2014-08-27

**Authors:** Martin Egelhaaf

**Affiliations:** 1 Department of Biology, Bielefeld University, Bielefeld, Germany

***General remarks***

The amount of information that is perceived by the peripheral visual system is thought to exceed the amount of information that can be processed by subsequent stages of the brain. Based on a host of experimental data mainly obtained with humans, both overt and covert strategies were concluded to facilitate the extraction of the behaviourally relevant information and to cope with the problem that the entire visual information cannot be processed at once. In previous studies by the same authors (or a subgroup of them) it was convincingly shown that honeybees (in contrast to bumblebees) employ mainly an overt serial strategy to search for previously learnt coloured target objects, if these are embedded between distracting cues. In analogy to similar results obtained with human observers, these conclusions were interpreted as being based on a kind of serial shift of the animals’ focus of attention. The analyses were based on the search performance (i.e. measuring both error rate and search time) while detecting a target object embedded in a variable number of distracting differently coloured objects.

The present study is based on a similar experimental design as employed in these previous studies and asks the highly interesting question of whether the visual system of honeybees is functionally regionalised with regard to a pattern detection/discrimination task. Honeybees were trained to detect a coloured target either in the upper or bottom row of a 3x3 matrix of possible object positions. In the test phase distracting objects were added in three different constellations to the stimulus matrix. Both target and distractor objects were placed either in the top row (‘dorsal group’) or only in the bottom row (‘ventral group’); in a ‘mixed group’, target and distractors could appear in the top row as well as in the bottom row. From the way the search performance improved under the different stimulus constellations, the authors concluded that honeybees flexibly adapt to a given foraging situation by focusing their search strategy to the location of the object within their visual field. The accuracy, decision time and flight paths of honeybees were concluded to depend on both the target’s location in the visual field and on the bees’ possibility to predict the target location. It is discussed whether honeybees use attentional mechanisms to increase their success rate in the detection task.

Although I am sympathetic with the overall goal of this project and agree with the authors’ overall conclusion, I have to admit that I am somewhat confused by parts of the experimental design of the study. Some further explanation of the rationale behind the analysis is likely to largely raise the potential of the study.

***Major issues***

After the training phase the bees could not have any *a priori* expectation with regard to target position, since target position was pseudo-randomized during the training procedure. Interestingly, a clear dorso-ventral asymmetry of the error rate of target detection was found even at the end of the training period. For the test phase the bees were subdivided into the three experimental groups that were tested with just one of the stimulus constellations. The rationale behind this design is not well explained and not immediately obvious. At least at first sight, one might have expected that the different constellations were tested again with all bees in a pseudo-random fashion.

One reason that this has not been done might have been the extreme dorso-ventral performance gradient after the training phase. Even without any distractor the coloured target could be detected with only a very high median error rate of 65% when presented in the top row. Surprisingly, no strong point is made that the detection performance is not much above chance level (this issue should have been tested statistically!), when the target was presented in the top row. Theoretically, this poor performance should not increase, if in the test phase – i.e. after adding two distractor objects - the target were also presented randomly in the top and bottom row of the stimulus arrangement. Hence, it might have been scientifically meaningful to ask whether the detection performance in the dorsal part of the visual field might increase, if the bee had the chance to build up an expectation that the target is not located ventrally. This might be a rationale for subdividing the bees into the three groups. Irrespective of whether or not this supposition is correct, an explanation for the experimental design should be given.

In any case, it can be concluded from the development of error rates, decision times, but also the trajectory data that the performance of the ‘dorsal group’ improves over time and that this finding is consistent with the interpretation that also dorsally located targets can be readily detected, if the bee has *a priori* knowledge that the target is likely to be found in an otherwise ‘suboptimal’ part of the visual field. Again, it might be helpful if this kind of conclusion were drawn explicitly in the paper. Whether this finding is just a consequence of a learning process or might be also linked to attentional mechanisms can then be discussed. However, it might be hard to conceptualise how attentional mechanisms can be pinpointed experimentally in the type of experiments that were performed in this study.

The hardest issue that remains to be explained are the data obtained with the ‘mixed group’ of bees. Although here no expectation can develop with regard of the location of the target, the performance improves over stimulus presentations to values that are much better than those obtained at the end of the training phase – even without any distractors (!). This highly surprising issue definitely needs to be discussed. This somehow apparent contradiction raises also the question whether – given a tremendous variability between bees but also between different flights of individual bees – the data base is sufficiently large to allow for drawing strong conclusions.  

***Minor issues***

*Title of the paper:*

Since the paper does not focus on the topic of attention, I would omit in the title of the paper the addendum “: a role for spatial attention?” Attention is just one possibility, in addition to several others, to explain the experimental results. Putting too much emphasis on attention already in the title may be somewhat misleading.

*Abstract*:

“Here we investigate if this asymmetry is caused by fixed search patterns or if bees can use alternative search mechanisms such as spatial attention, which allows flexible focusing on different areas of the visual field.” I do not see that the investigation of this alternative has been the focus of the experimental analysis of the study. At least the results do not allow answering this question in an unambiguous way.

*Introduction:*

*1 ^st^ paragraph*: It appears to be surprising that the paper starts by considering whether the distinct spatial asymmetry in colour and pattern learning of honeybees as observed in previous studies might be a consequence of specialisations in eye morphology, as has been proposed for detecting the queen by the drone bee eye. It should be noted that this sort of detection task is conceptually very different: in the drone bee case we are dealing with detection thresholds where - for obvious reasons - spatial acuity and sensitivity, i.e. anatomical and physiological retinal parameters, are the relevant factors, because they determine at which distance a small target (i.e. in this case the queen) can be seen. In the learning experiments the targets were usually sufficiently large, and no spatial resolution or sensitivity problem existed for them to be detected.

*Methods:*

- What was the frame rate and resolution of the camera system?
- “The stimuli subtended a visual angle of …” (and following sentence): The authors probably mean the individual discs. “stimuli” not specific enough.
- It should be explained why a virtual decision line was chosen. It might have been more intuitive to monitor the landing on the different targets. Would the error rate change, if the landings were recorded rather than the crossing of a virtual decision line?
- What happened to the bees after the individual training and test flights? Do they return to the entrance hole of the flight arena or were they caught and ‘manually’ bought back to their hive?
- Were the individual bees labelled and could be identified? This might have been a precondition to be able to monitor changes in detection performance.
- What about the orientation of body and head in 3D space? How well can these parameters be resolved? This is important information, especially if the retinal position of the objects needs to be assessed. The term ‘flight angle’ should be explained.
- “We used mixed linear models to test the effect of treatment group, target location and learning on the error rate and on the decision time, respectively. Furthermore, the identity of the individual bees was implemented into the model as a random factor.” It is not entirely clear what these sentences really mean. ‘Mixed linear models’ and ‘implementing individual bees into a model as a random factor’ should be explained.

*Results:*

- What does ‘overall error rate’ mean?
- Figure 3: If I understand the explanation of the figure correctly, the data point ‘6’ ‘N° of flights’ shown in A and B should be the mean of the corresponding data points ‘3’ and ‘6’ ‘N° of flights’ in C. Since in C both ‘dorsal group’ data points have a larger value than the corresponding data point in B, this interpretation cannot be correct – or something went wrong with the data. Please check.
- Legend of figure 4: “Please note that in the mixed group the flight number refers to number of trials absolved with the particular target situation and not to the total number of trials the bee has absolved”. This statement is somewhat cryptic. Please explain.

*Discussion:*

Most issues concerning the Discussion have already been mentioned above. Here just some minor, though relevant details:

- During the flights towards the target bees change their height. How is this done? Do they change the pitch angle of their body and, in particular, of the head when changing height as compared to the pitch angle during horizontal flights? This is important, since it pertains to the retinal area with which the target is seen. *Drosophila*, for instance, changes the pitch angle of its body when changing height, whereas it keeps the head orientation in space relatively constant (by changing the head angle relative to the body).
- “… bees could have increased the area of their attentional focus to a size large enough to cover both rows”. Obviously, by assuming such an attentional process almost every change in detection performance can be explained. However, from an epistemological point of view such a hypothesis makes only sense, if an experiment could be designed by which this hypothesis can be tested.

I have read this submission. I believe that I have an appropriate level of expertise to confirm that it is of an acceptable scientific standard, however I have significant reservations, as outlined above.

# Peer review: Referee response for version 1

**DOI:** 10.5256/f1000research.5123.r5787

**Published:** 2014-08-26

**Authors:** Bruno van Swinderen

**Affiliations:** 1 Queensland Brain Institute, University of Queensland, Brisbane, QLD, Australia

In this study Morawetz, Chittka, and Spaethe present results from a behavioral paradigm for honeybees where they examine spatial attention. Bees were trained to select a color, using classical conditioning with sugar versus quinine. Trained bees were then presented with various scenarios whereby the targets were presented together with distracters, either in the upper or lower visual field. The authors find that targets in the ventral field led to a more efficient search strategy. Importantly, it appears that honeybees form expectations about where their target will be, in repeated flights.

These are interesting results, which help consolidate the view that insects display a selective attention. In particular, the data supporting the view that there is an expectation already formed early in the flight angle is very intriguing. It is nice to see a paper where the behavior itself is examined closely, rather than only the proportion of choice outcomes. This helps to better show how attention might be operating in these simple animals.

The paper is close to indexation quality. There are a couple questions that might need to be attended to, to further improve the quality of the work.

First, it is quite unclear how the position of the “decision line” was arrived at. While it is evidently depending on previously published work, one concern is that varying the position of this decision line might substantially change the results and conclusions. The decision line is currently 5cm away from the end wall, and both metrics (error rate and decision time) depend on this distance. Clearly, a bee flying downwards is exploiting gravity to get to the decision line, while a bee flying upwards is fighting gravity. This simple factor could account for the different flight dynamics seen for either situation (as is evident in the informative single examples shown in Figure 5). Can the authors exclude a non-trivial explanation here for the effects of gravity? With regard to the error rate for example, it is not clear whether a “mistake” is meaningful here. Maybe bees fly more erratically upwards (they do). More generally, it would be good to have a bit more of a rationale for why this 5cm point was chosen. How different would the results have been at 7cm or 10cm?

Second, the error rate metric does not seem to account for proximity. Thus, a bee crossing the decision line close to the target is as equally wrong as a bee crossing a whole square away? This seems strange. Should the error rate be weighted in some way? How might that change the results?

Minor:

Sareen et al (2011) showed that fruit fly attention is mostly ventral. This might be discussed a bit more, in connection to the current findings in the bee.

I have read this submission. I believe that I have an appropriate level of expertise to confirm that it is of an acceptable scientific standard.
